# Whole-genome variants dataset of 209 local chickens from China

**DOI:** 10.1038/s41597-024-02995-w

**Published:** 2024-02-05

**Authors:** Xiaodong Tan, Jiawen Zhang, Jie Dong, Minjie Huang, Qinghai Li, Huanhuan Wang, Lijuan Bai, Ming Cui, Zhenzhen Zhou, Shuyuan Yang, Deqian Wang

**Affiliations:** 1 Institute of Animal Husbandry and Veterinary Science, Zhejiang Academy of Agricultural Sciences, Hangzhou, 310021 China; 2 Animal Husbandry Institute, Hangzhou Academy of Agricultural Sciences, Hangzhou, 310024 China; 3 Zhejiang Animal Husbandry Technology Extension and Breeding Livestock and Poultry Monitoring Station, Hangzhou, 310020 China

**Keywords:** Animal breeding, Comparative genomics, Genetic markers

## Abstract

Compared to commercial chickens, local breeds exhibit better in meat quality and flavour, but the productivity (e.g., growth rate, body weight) of local chicken breeds is rather low. Genetic analysis based on whole-genome sequencing contributes to elucidating the genetic markers or putative candidate genes related to some economic traits, facilitating the improvement of production performance, the acceleration of breeding progress, and the conservation of genetic resources. Here, a total of 209 local chickens from 13 breeds were investigated, and the observation of approximately 91.4% high-quality sequences (Q30 > 90%) and a mapping rate over 99% for each individual indicated good results of this study, as confirmed by a genome coverage of 97.6%. Over 19 million single nucleotide polymorphisms (SNPs) and 1.98 million insertion-deletions (InDels) were identified using the reference genome (GRCg7b), further contributing to the public database. This dataset provides valuable resources for studying genetic diversity and adaptation and for the cultivation of new chicken breeds/lines.

## Background & Summary

Chickens are among the most important farm animals supplying eggs, meat, and other products to humans. After long-term domestication and human-driven selection, hundreds of distinctive chicken breeds are now cultivated worldwide, and chicken meat has been the largest meat resource since 2019^1^. In particular, more than one hundred local breeds have been identified in China, constituting almost half of the broiler market. Compared to commercial broilers (e.g., Arbor Acre, Ross, and Cobb), local chickens exhibit markedly improved meat quality and flavour, adaptation to the environment, and disease resistance^2,3^. However, local breeds have not undergone intense artificial selection for productivity traits, which may explain their lower production performance compared to commercial breeds^4,5^. However, compared to pigs or cattle, chickens are more efficient and environmentally friendly livestock. Their feed conversion ratio is ten times that of cattle, and the carbon emissions of broilers are only 1/10 of those of cattle^6^. Therefore, it is necessary to investigate genomic markers and genetic mechanisms related to economic traits via whole-genome sequencing to bridge the gap in production performance and accelerate breeding progress^7^.

Simultaneously, domestic chickens are desirable models for investigating genetic adaptation and diversity and disease-related markers due to the advantages of their short reproductive cycle, small body size, and identical ancestors. After domestication from red jungle fowl (*Gallus gallus spadiceus*)^8^, chickens were cultivated with the aim of meeting various human demands, and meat-type (e.g., Xiaoshan chicken), egg-type (e.g., Xianju chicken), ornamental-type (e.g., Silkie chicken), and game fowl breeds (e.g., Luxi gamecocks, Henan gamecocks) were developed^9–12^, as well as the commercial broilers (e.g., broiler line B, Cobb)^13^. Therefore, it is relatively easy to unambiguously infer the driving factors of phenotypic or behavioural changes in these chickens. Additionally, domestic chickens can be used to generate special populations, such as F2 populations^14,15^ and advanced intercross lines^16^, which contributes to exploring the genetic mechanisms of chicken complex traits and provides new insights into genomic breeding.

Genomic analysis can reveal the demographic history of different chicken breeds and reconstruct gene flow among them, which contributes to a better understanding of the domestic history and potential mechanisms of some economic traits, such as breast muscle yield^17^, yellow skin^4,18^. The analysis of genome-wide variants in chickens distributed in different regions can be used to investigate genetic adaptation and diversity, especially adaptation to environmental conditions, such as altitude, temperature, and anoxic environments^19–22^. Moreover, selective sweep analysis based on single nucleotide polymorphisms (SNPs) is an effective method for identifying genetic markers and mechanisms underlying chicken production performance, reproduction, immunity, etc. This approach provides important insights into modern breeding systems^20–25^. In addition to SNPs and insertion‒deletions (InDels), genome-wide sequencing has also been used to identify copy number variants (CNVs)^26^ and structural variants (SVs)^27^, although the detection rate and efficiency of SV calling are relatively low compared to those of PacBio sequencing^28^. Therefore, genomic analysis using the whole-genome variants of local chickens is an effective approach for elucidating genetic diversity and selective signatures during long-term domestication.

This study provides a whole-genome sequencing dataset from hundreds of local chickens (n = 209) of 13 local chicken breeds in China, including meat-type, egg-type, and ornamental-type chickens. A total of more than 19 million SNPs and 1.98 million InDels were identified on chromosomes 1–39 and one sex chromosome (Z) by aligning the sequencing reads against the most recent reference genome of chickens (assembly bGalGal1.mat.broiler. GRCg7b, GCF_016699485.2). The summarization of high-quality sequences (Q30 > 90%, mapping rate > 99%) confirmed the high accuracy and resolution of this genetic analysis. This dataset is expected to be useful in many studies, such as those aimed at identifying putative regions of positive selection, inferring demographic history, analysing gene flow, detecting candidate genes related to economic traits, determining genetic adaptation to local environmental factors, discovering breed-specific variants or markers, analysing genetic diversity, or developing SNP genotyping arrays for use in chicken breeding systems or species identification. Moreover, the whole-genome variants of most chicken breeds (except for Beijing You and Silky-feather chickens) included in the present study have not been reported. Therefore, this dataset provides an ideal resource for population genetics and evolutionary analyses. Furthermore, this database represents an important supplement to the chicken variant database and plays a crucial role in reconstructing the demographic and domestication history of chickens.

## Methods

### Sampling

Blood sampling from 13 chicken breeds was performed in Zhejiang Province, China. The following breeds were included (Supplement Table S1): BE, Baier chicken; BJY, Beijing You chicken; DXB, Dongxiang Black chicken; JSW, Jiangshan white-feathered chicken; LH, Luhua chicken; LY, Longyou chicken; SF, Silky-feather chicken; SYJ, Songyang Jin chicken; WL, Wenling chicken; XJ, Xianju chicken; XS, Xiaoshan chicken; XX, Xiaoxiang chicken; and YD, Yandang chicken. We collected a 1 ml blood sample from the wing vein of each individual and stored it in a 2 ml anticoagulation tube at −20 °C. All procedures associated with the chickens used in this study were consistent with the standards of the Laboratory Animal Guidelines for the Ethical Review of Animal Welfare and were approved by the Animal Use Committee of Zhejiang Academy of Agricultural Sciences (No. 2022ZAASLA68).

### Genomic DNA extraction and quality control

**Fig. 1:**
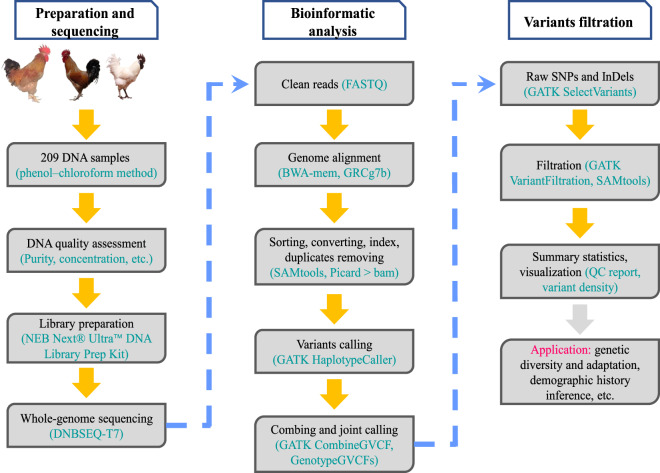
The workflow of the library preparation, sequencing, genome mapping, and variant calling and filtration. The pipeline was consistent with the GATK recommended protocol for variant identification.

The workflow from sampling to variant filtration is shown in Fig. 1. Genomic DNA was extracted from blood samples using the phenol‒chloroform method. DNA quality control was performed as follows: 1) DNA degradation and contamination were monitored on 1% agarose gels; 2) the OD 260/280 ratio was determined with a NanoDrop instrument to check the purity of the DNA; and 3) the DNA concentration was measured with a Qubit® DNA Assay Kit on a Qubit® 3.0 Fluorometer (Invitrogen, USA). Finally, more than 0.2 μg of DNA fragments with no degradation or contamination and an OD value of 1.8~2.0 were used for library construction.

### Library preparation and sequencing

A sequencing library was created with the NEB Next UltraTM DNA Library Prep Kit for Illumina (NEB, USA). A mean size of 350 bp was achieved by shearing the genomic DNA. Before sequencing, the DNA fragments were subsequently submitted to end polishing, A-tailing, and adapter addition. PCR amplification and purification were then carried out using an AMPure XP system (Beckman Coulter, Beverly, USA). Using an Agilent 2100 Bioanalyzer and Qubit 3.0 Fluorometer (Invitrogen, USA), the quality of the library was evaluated based on the insert size and DNA concentration. Real-time PCR (>2 nM) was used to quantify the results. Ultimately, a flow cell containing the qualifying DNA nanospheres was filled, and a DNBSEQ-T7 platform was used for sequencing.

### Data quality control, mapping, and variant calling

Clean reads were produced by trimming the raw reads using FASTP v0.21 software^29^, and reads meeting any of the following conditions were discarded: 1) one read of a pair contained adapter contamination (>10 nucleotides aligned to the adapter, allowing ≤10% mismatches); 2) more than 10% of bases were uncertain in either read; and 3) the proportion of low-quality (Phred quality <5) bases was over 50% in either read. The trimmed clean reads were evaluated by FastQC v0.12.1 software. Then, the clean reads were aligned against the most recent chicken reference genome (GRCg7b, GCF_016699485.2) using the Burrows‒Wheeler Aligner (BWA) v0.7.17 with the MEM algorithm^30^. The results were sorted, indexed, and converted to BAM files using SAMtools v1.12 software^31^. After removing polymerase chain reaction (PCR) duplicates, SNP calling was performed using the joint calling strategy, realized with the HaplotypeCaller, CombineGVCFs, and GenotypeGVCFs functions in the Genome Analysis Toolkit (GATK) v4.2.2^32^. Next, we removed SNPs using GATK v4.2.2 according to specific standards: QualByDepth <2.0, FisherStrand >40.0, StrandOddsRatio >3.0, MappingQuality <40.0, and ReadPosRankSum <−8.0. A total of 19,820,641 biallelic SNPs and 1,980,710 biallelic InDels identified on autosomes after further quality control using VCFtools v0.1.13^33^ (--max-alleles 2 --min-alleles 2 --min-meanDP 3 --maf 0.01) were retained for subsequent analysis. The missing alleles were imputed by Beagle v5.1 software^34^ with the default parameters.

### Variant annotation

The identified variants were annotated using ANNOVAR software^35^, the SNPs/InDels were classified according to the genomic location, such as exonic, intergenic, downstream, upstream, and others.

### Variant density, genetic diversity, and polymorphism information content (PIC) estimation

The variant density was calculated as the ratio of the total length of the genome (with N bases removed) to the variant number. The SNP/InDel density was calculated and visualized with the CMplot package^36^. The genetic diversity (π) was calculated using VCFtools v0.1.13^33^ with window size of 50 kb. The PIC was estimated using the following formula: PIC = 1 − *p*_*i*_^2^ − *q*_*i*_^2^, where *p*_*i*_ indicates the frequency of the minor allele of SNP_i_ and *q*_*i*_ indicates the frequency of the major allele of SNP_i_.

## Data Records

Raw FASTQ files for whole-genome sequencing were deposited in the NCBI Sequence Read Archive (SRA) and have been assigned BioProject accession number PRJNA942350 (https://identifiers.org/ncbi/insdc.sra:SRP426730)^37^. The raw VCF file containing SNPs and InDels was deposited in the EVA database with accession number PRJEB71347 (https://identifiers.org/ena.embl:PRJEB71347)^38^. The quality control results of raw reads and annotation files of variants have been deposited in the the Figshare database with the following digital object identifier: 10.6084/m9.figshare.24751956.v2^39^. The relationship between the chicken ID in the VCF files and the SRA database was shown in Supplement Table S2.

## Technical Validation

### Quality control of sequencing results

For each individual, an average of 17.3~28.5 Gb of raw data from were obtained, with 91.4% of the data achieving a Phred quality score of 30 (indicating sequencing accuracy of 99.9%) on average (Table 1). A stable GC content (42.6%) was demonstrated for the sequence (Table 1, Supplement Table S3). A genome mapping rate greater than 99% and an average genome coverage of 97.1% (with N bases removed) were obtained (Table 2, Supplement Table S4).

**Table 1:** Summary statistics for quality control of sequences^1^.

	Raw Data (G)	Clean Data (G)	Error Rate (%)	Q20 (%)	Q30 (%)	GC Content (%)
BE	19.0	18.9	0.03	97.7	92.5	41.7
BJY	21.6	21.6	0.03	97.3	91.6	42.4
DXB	24.5	24.5	0.03	97.6	92.1	43.4
JSW	27.2	27.2	0.03	97.3	91.8	43.0
LH	23.5	23.4	0.03	97.6	92.3	43.8
LY	17.3	17.3	0.03	96.2	88.3	41.3
SF	23.7	23.6	0.03	96.7	89.5	41.3
SYJ	21.6	21.5	0.03	97.3	91.8	42.7
WL	28.5	28.5	0.03	97.0	90.7	42.4
XJ	22.2	22.1	0.03	97.3	91.9	44.0
XS	23.9	23.8	0.03	97.9	93.1	42.7
XX	24.0	24.0	0.03	97.3	91.8	43.4
YD	20.9	20.9	0.03	97.0	91.2	42.0
Total^2^	22.9	22.9	0.03	97.2	91.4	42.6

^1^All the terms below were calculated in each chicken breed; values represent means over individual sequences per breed.

^2^Indicated all terms were calculated in all individuals and presented in average value.

**Table 2:** Summary statistics for genome alignment analysis^1^.

	Clean reads	Mapped reads	Mapping rate (%)	Coverage (1×, %)^2^	Coverage (4×, %)^3^
BE	126,170,232	125,470,548	99.4	98.1	96.8
BJY	143,657,126	142,671,245	99.3	98.5	97.3
DXB	163,128,537	161,925,595	99.3	98.5	97.3
JSW	181,380,251	180,053,806	99.3	98.6	97.6
LH	156,255,165	154,983,029	99.2	98.5	97.5
LY	115,382,337	114,778,980	99.5	97.8	96.0
SF	157,537,327	156,820,634	99.5	97.9	96.6
SYJ	143,553,599	142,616,427	99.3	98.6	97.4
WL	190,025,969	188,943,315	99.4	98.5	97.3
XJ	147,572,126	146,239,584	99.1	98.7	97.4
XS	158,809,516	157,845,199	99.4	98.5	97.5
XX	159,689,655	158,416,884	99.2	98.5	97.3
YD	139,173,234	138,280,098	99.4	98.0	96.9
Total^4^	152,487,313	151,465,026	99.3	98.4	97.1

^1^All the terms below were calculated in each chicken breed; values represent means over individual sequences per breed.

^2^The coverage (1×) was calculated by the ratio of total length of mapped reads (mapped by at least 1 read) to the genome length.

^3^The coverage (4×) was calculated by the ratio of total length of mapped reads (mapped by at least 4 reads) to the genome length.

^4^Indicated all terms were calculated in all individuals and presented in average value.

### Filtration of SNP and InDel datasets

**Fig. 2:**
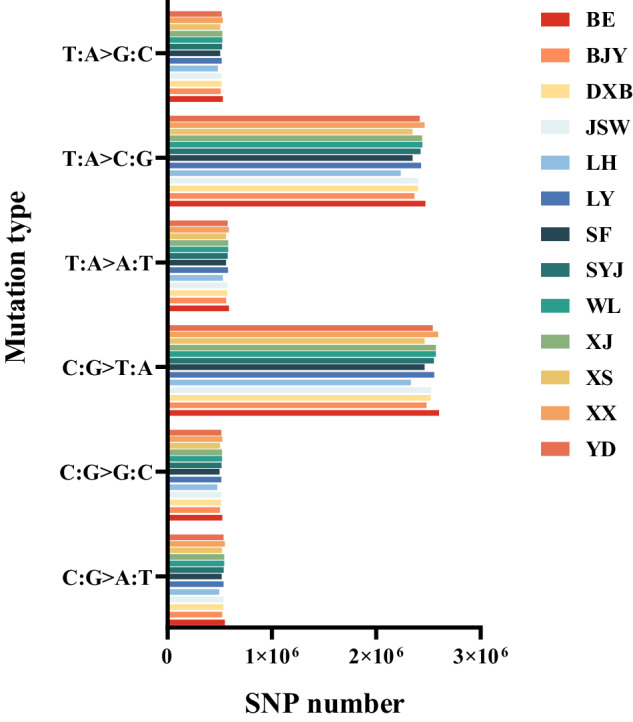
Statistics for the SNP number of different mutation types.

**Fig. 3:**
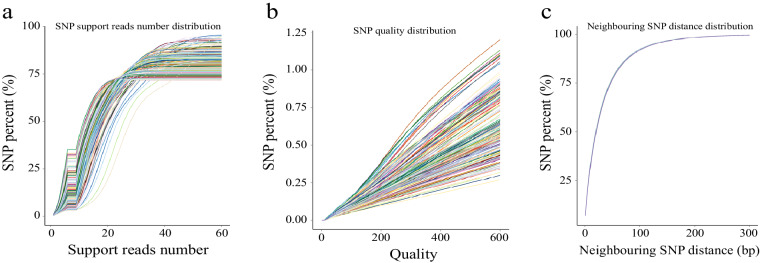
Statistics for the SNP percent in support reads number (**a**), quality (**b**), neighbouring SNP distance (**c**). The different colour indicated the various individual.

The joint calling strategy was used in this procedure, and a total of 27 million raw SNPs and 2.75 million raw InDels were identified in the population of 13 chicken breeds. To exclude low-quality variants, we used the VariantFiltration function in GATK software^32^. The specific standards used are described in the Methods section above. After the first round of quality control, the SNPs and InDels were further filtered using VCFtools v0.1.13 software^33^, and only biallelic variants with a minor allele frequency of 0.01 and a minimum sequencing depth of 3 were retained. We calculated statistics for SNP types, and T:A > C:G-type mutations were mainly identified in this population (Fig. 2). Figure 3 shows the relationships among SNP quality, supported read number, SNP percentage, and neighbouring SNP distance. We detected a positive correlation between SNP quality and percentage, and most SNPs were supported by at least 20 reads (Fig. 3a,b). This indicates the high quality of the identified SNPs.

### Summary statistics of SNPs and InDels across the whole genome

**Fig. 4:**
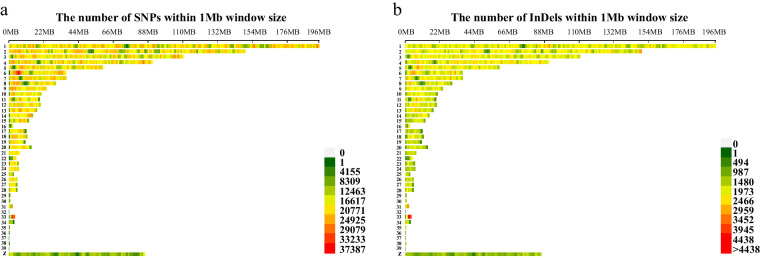
Distribution of SNP and InDel across the whole-genome of 13 local chicken breeds. (**a**) SNP density statistics across the whole-genome. (**b**) InDel density statistics across the whole-genome.

**Fig. 5:**
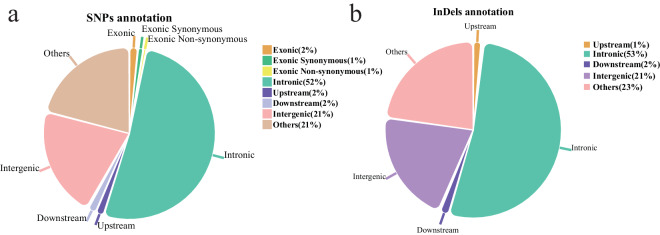
Annotation result for SNPs (**a**) and InDels (**b**).

**Fig. 6:**
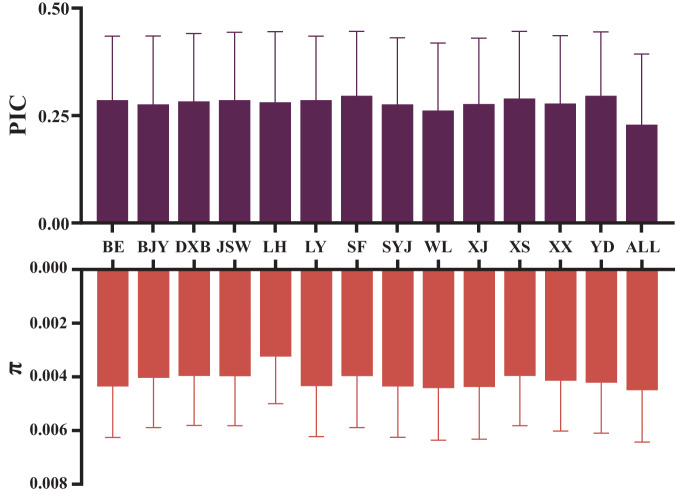
Estimation of genomic PIC (**a**) and π (**b**) based on SNPs of 13 chicken breeds.

The high-quality SNPs and InDels were distributed across the genome with an average density of 1 SNP every 52 bases and 1 InDel every 521 bases (Table 3). Figures 3c, 4a show the high density of SNPs across the whole genome. The density of InDels was relatively low (Fig. 4b). Chromosomes 16, 29–32, and 34–38 were defined as dot chromosomes, while chromosomes 22, 25 and 33 were defined as microchromosomes^40^. We found that the SNP density on these chromosomes exhibited a polarized distribution (Table 3). Based on variants annotation, SNPs/InDels were mainly distributed in intronic and intergenic regions (Fig. 5). And more SNPs rather than InDels were located in exons (Fig. 5a). The PIC and genetic diversity π of each breed are shown in Fig. 6, the genomes of the SF and YD chickens exhibited higher polymorphism, and LH chickens were found significant lower genetic diversity.

**Table 3:** Summary statistics of SNPs and InDels in each chromosome ^1^.

	Chromosome ID in RefSeq	Size (Mb)	SNP number	SNP density (bp/SNP)	InDel number	InDel density (bp/InDel)
1	NC_052532.1	196.5	3,792,180	51.8	387,962	506.4
2	NC_052533.1	149.5	2,843,003	52.6	298,494	501.0
3	NC_052534.1	110.6	2,110,715	52.4	226,020	489.5
4	NC_052535.1	90.9	1,775,144	51.2	194,660	466.8
5	NC_052536.1	59.5	1,152,021	51.7	123,204	483.0
6	NC_052537.1	36.2	780,677	46.4	77,277	468.7
7	NC_052538.1	36.4	720,446	50.5	74,358	489.3
8	NC_052539.1	29.6	533,582	55.4	53,421	553.7
9	NC_052540.1	23.7	489,580	48.5	47,935	495.0
10	NC_052541.1	20.5	402,717	50.8	39,066	523.5
11	NC_052542.1	19.6	344,122	57.1	35,740	549.5
12	NC_052543.1	20.1	422,745	47.6	40,912	491.8
13	NC_052544.1	17.9	378,719	47.3	34,567	518.1
14	NC_052545.1	15.3	311,325	49.2	29,299	523.2
15	NC_052546.1	12.7	228,845	55.5	21,262	597.3
16	NC_052547.1	2.7	78,320	34.6	4,793	565.4
17	NC_052548.1	11.1	238,107	46.6	18,736	591.9
18	NC_052549.1	11.6	239,971	48.4	20,398	569.7
19	NC_052550.1	10.5	198,804	52.6	16,767	623.8
20	NC_052551.1	14.3	275,359	51.8	24,320	586.8
21	NC_052552.1	7.0	136,143	51.2	11,666	597.5
22	NC_052553.1	4.7	64,259	73.0	5,891	796.1
23	NC_052554.1	6.3	124,025	50.4	10,355	603.6
24	NC_052555.1	6.5	136,856	47.3	10,697	605.8
25	NC_052556.1	3.1	62,194	49.4	6,535	469.8
26	NC_052557.1	5.4	118,467	45.2	9,778	547.1
27	NC_052558.1	5.2	116,884	44.7	10,115	517.1
28	NC_052559.1	5.4	106,902	50.9	9,777	556.4
29	NC_052560.1	0.7	23,083	31.6	1,314	555.6
30	NC_052561.1	0.8	11,353	66.9	1,635	464.8
31	NC_052562.1	2.5	135,679	18.1	6,636	370.7
32	NC_052563.1	0.1	3,420	38.0	244	532.8
33	NC_052564.1	3.8	225,141	17.1	11,028	348.2
34	NC_052565.1	3.5	47,280	73.4	5,514	629.3
35	NC_052566.1	0.6	13,799	39.9	1,770	310.7
36	NC_052567.1	0.4	6,534	55.1	1,151	312.8
37	NC_052568.1	0.2	3,745	42.7	187	855.6
38	NC_052569.1	0.7	7,578	88.4	1,457	459.8
39	NC_052570.1	0.2	2,594	69.4	412	436.9
Z	NC_052572.1	86.0	1,158,323	74.3	105,357	816.7
Total	1,032.0	19,820,641	52.1	1,980,710	521.0	

^1^The variant density was calculated by the ratio of chromosome length to variant number.

## Usage Notes

Whole-genome sequencing allows us to obtain the SNPs and InDels across the whole genome using the bioinformatic pipeline of this study. However, the CNVs and SVs were not included in the current study. As same as the previous report in human and chickens^8,41–43^, we did not consider the ploidy of variants in sex chromosome ZW. And the variants in chromosome W were discarded due to the uncertain gaps in chromosome. Although SNPs are more widely used for investigating investigate the genetic diversity and dissecting the genetic mechanism of economic traits, the heritability of traits of interest cannot be fully explained by SNPs, namely, missing heritability. These traits are also influenced by epigenetic factors. The effect of missing heritability could be relieved by increasing the sample size and sequencing depth and by considering the epistatic effects and SVs and CNVs, which also contributed to the identification of additional novel loci^44^. Additionally, we aligned the sequencing data to the reference genome (GRCg7b, GCF_016699485.2), which was assembled based on one broiler and did not include all the variants in the reference genome. Therefore, the data produced from this study may produce an incomplete explanation of the genetic background due to the missing alignment. Overall, our data provide insights into the evaluation of chicken population structure, and are an efficient dataset for identifying the CNVs and SVs. These data can be used in the construction of chicken pangenomes or graph pangenomes together with the Pacbio and Oxford Nanopore Technology data.

### Supplementary information(The order of supplementary table should be changed, the table S1 should be arranged to the front)

Supplementary Table S1

Supplementary Table S2

Supplementary Table S3

Supplementary Table S4

## Data Availability

The variant calling procedure was conducted in accordance with the standard bioinformatic workflow recommended by GATK, and all the steps were performed in the CentOS system. The detailed codes and parameters used in this study are provided as follows: **(1) Quality control for the raw reads:** **Software:** FASTP v0.21, FastQC v0.12.1 **Code for trimming:** fastp -I ${read1} -I ${read2} -o ${read1.qc} -O ${read2.qc} -q 5 -g -u 50 -n 15 -l 150 --min_trim_length 10 --overlap_diff_limit 1 --overlap_diff_percent_limit 10 **Code for quality control**: Fastqc -f fastq -t 6 -o ${sample.qc} ${read1.qc} ${read2.qc} **(2) Genome alignment:** **Software:** BWA-mem v0.7.17 **Code:** bwa mem -t 10 -M -R “@RG\tID:${individual}\tLB:${individual}\tPL:illumina\tSM:${individual}” /genome.index/bwa.index/gga7.${read1.qc} ${read2.qc} > ${individual}.sam **(3) Sorting, files converting, and indexing:** **Software:** SAMtools v1.12 **Code for sorting and file converting:** samtools sort -m 10 G -S ${individual}.sam -o ${individual}.sorted.bam -@ 10 **Code for bam file indexing:** samtools index -b ${individual}.sorted.bam ${individual}.sorted.bai **(4) Statistics for sequencing depth:** **Software:** Mosdepth v0.2.9 **Code:** mosdepth -t 6 ${individual.depth} ${individual.bam} **(5) Removing the duplicates** **Software:** Picard v2.26 **Code:** java -Xms100g -Xmx200g -jar picard.jar MarkDuplicates INPUT = ${individual}.sorted.bam OUTPUT = ${individual}.rmdup.bam M = ${individual}.metrices.txt REMOVE_DUPLICATES = true CREATE_INDEX = true **(6) Variants calling** **Software:** GATK v4.2.2 **Code for the GVCF model generated using HaplotypeCaller:** gatk --java-options “-Xmx60g -Xms20g” HaplotypeCaller --native-pair-hmm-threads 80 -R Gallus_gallus.bGalGal1.mat.broiler.GRCg7b.dna_sm.toplevel.fa -I ${individual}.rmdup.bam -ERC GVCF -O ${individual}.g.vcf **Code for combining GVCF files:** gatk --java-options “-Xmx80g -Xms60g” CombineGVCFs -R Gallus_gallus.bGalGal1.mat.broiler.GRCg7b.dna_sm.toplevel.fa --variant individual1.g.vcf --variant individual2.g.vcf --variant individual3.g.vcf -O merge.g.vcf **Code for variant joint calling:** gatk --java-options “-Xmx80g -Xms60g” GenotypeGVCFs -R Gallus_gallus.bGalGal1.mat.broiler.GRCg7b.dna_sm.toplevel.fa -V merge.g.vcf -O merge.vcf **(7) Variants extraction and hard filtration:** **Software:** GATK v4.2.2.0 **Code for SNP extraction:** gatk --java-options “-Xmx80g -Xms60g” SelectVariants -V merge.vcf -O raw.snp.vcf --select-type-to-include SNP **Code for InDel extraction:** gatk --java-options “-Xmx80g -Xms60g” SelectVariants -V merge.vcf -O raw.indel.vcf --select-type-to-include INDEL --max-indel-size 50 **Code for filtration:** gatk --java-options “-Xmx80g -Xms60g” VariantFiltration -R Gallus_gallus.bGalGal1.mat.broiler.GRCg7b.dna_sm.toplevel.fa -V raw.snp.vcf -filter-expression QD < 2.0 || FS > 40.0 || SOR > 3.0 || MQ < 40.0 || ReadPosRankSum < −8.0” --filter-name filter -O filtered.snp.vcf **(8) Further filtration:** **Software:** VCFtools v0.1.13 **Code:** vcftools --vcf filtered.snp.vcf --max-alleles 2 --min-alleles 2 --min-meanDP 3 --remove-filtered-all –recode --out qc.snp **(9) Genotypes imputation:** **Software:** Beagle v5.1 **Code:** java -Xmx100g -Xms50g -jar beagle.jar gt = qc.snp.vcf impute = true nthreads = 20 out = qc.phased.snp
